# Rcl1 suppresses tumor progression of hepatocellular carcinoma: a comprehensive analysis of bioinformatics and in vitro experiments

**DOI:** 10.1186/s12935-022-02533-x

**Published:** 2022-03-09

**Authors:** Yu Jiaze, Hou Sinan, Yang Minjie, Zhou Yongjie, Du Nan, Wang Liangwen, Zhang Wen, Luo Jianjun, Yan Zhiping

**Affiliations:** 1 Department of Interventional Radiology, Zhongshan Hospital, Fudan University, 180 Fenglin Road, Shanghai, 200032 China; 2 National Clinical Research Center for Interventional Medicine, 180 Fenglin Road, Shanghai, 200032 China; 3 Shanghai Institution of Medical Imaging, 180 Fenglin Road, Shanghai, 200032 China

**Keywords:** Rcl1, HCC, rRNA processing factor, Cell cycle, Immune cells infiltration

## Abstract

**Background:**

RNA 3’-terminal phosphate cyclase-like protein (Rcl1) is involved in pre-rRNA processing, but its implication in cancers remains unclear.

**Methods:**

*RCL1* expressions in 21 malignancies was examinated through GEPIA website portal. Clinical implication data related to *RCL1* level in Hepatocellular Carcinoma (HCC) samples were downloaded through TCGA, ICGC, GEO databases. Survival analysis and gene function enrichment analyses were performed through R software. The correlation between *RCL1* expression and tumor immune infiltration was assessed via the TIMER2.0 database. The effects of Rcl1 overexpression or knockdown on cell growth and metastasis was evaluated by CCK8, transwell, and cell cycle assays.

**Results:**

*RCL1* expression is commonly down-regulated in HCC. The lower expression of *RCL1* is associated with higher tumor stage, higher AFP level, vascular invasion, and poor prognosis. *RCL1* expression has a significant correlation with immune cells infiltration in HCC, especially myeloid-derived suppressor cell (MDSC). Moreover, it was further identified that Rcl1 expression was reduced in HCC cell lines and negatively correlated with invasion of HCC cell lines. Immunofluorescence (IF) analysis revealed that the level of Rcl1 expression in the cytoplasm of HCC cells is significantly lower than that in the cytoplasm of L-02 cell. Moreover, both gain- and loss-of-function studies demonstrated that Rcl1 inhibited the growth and metastasis of HCC cells and regulated cell cycle progression in vitro.

**Conclusions:**

Rcl1 may serve as a novel tumor suppressor in HCC, and its biological effect needs further study.

**Supplementary Information:**

The online version contains supplementary material available at 10.1186/s12935-022-02533-x.

## Highlight

Rcl1 mRNA expression is down-regulated within HCC tissues and associated with poor prognosis and disease progression. Anti-cancer effects of Rcl1 on HCC were confirmed in vitro. Rcl1 may be a potential tumor suppressor in HCC.

**Supplementary Information:**

The online version contains supplementary material available at 10.1186/s12935-022-02533-x.

## Introduction

Primary liver cancer, the 6th most prevalent malignancy in the world, is the third leading cause of cancer-associated death worldwide [1]. Hepatocellular carcinoma (HCC) is the most common subtype of primary liver cancer (75–85%) [2]. Despite the large numbers of effective treatments of mid-advanced HCC exist, the long-term prognosis still remains poor. Therefore, lucubrating on the molecular mechanism of HCC development could contribute to the identification of new therapeutic targets.

Ribosome had initially been considered as a completely homogenous cellular organelle that simply synthesize the protein. However, ribosome heterogeneity was suggested having as evidence the findings that the ribosome proteome, ribosome gene transcriptome, and ribosome biogenesis factors differ between cells and tissues [3, 4]. Besides, the different underlying causes of ribosomopathies and their tissue-specific phenotypes also pointed out the variation in the ribosome composition and function [5, 6]. Furthermore, many studies have showed that the widespread deficiency in ribosome function and regulation of ribosome activity by oncogenes both could promote cancer development and progression [7]. Furthermore, it was reported that ribosome composition, maturation, and function could promote in the cancer chemo-resistance [8, 9]. In particular, many agents targeted into ribosome could be sensitive to several cancers that had not responded to chemotherapy [10, 11]. Therefore, studying the role of ribosome in cancer development becomes a necessity.

*RCL1* encodes the RNA 3′-terminal phosphate cyclase-like protein, a number of RNA cyclase families but without cyclase activity [12]. In a yeast study, it was suggested that Rcl1p could serve as an endonuclease that affects the cleavage steps in the 5'-external transcribed spacer and internal transcribed spacer-1 regions of the ribosomal RNA precursor [13, 14]. However, the endonuclease activity of Rcl1 in human cells is controversial [15]. Minguez et al. [16] reported that Rcl1 mRNA expression was associated with vascular invasion of HCC through transcription sequencing. Until now, the role and the exact mechanisms of Rcl1 in HCC development still remains unclear.

In the present study, we explored the mRNA expression and the clinical implications of Rcl1 in HCC patients by using several HCC cohorts. Moreover, the effects and mechanisms of Rcl1 in HCC cell line was further studied through in vitro experiments.

## Materials and methods

### Data resource

*RCL1* expression in various cancer and its relationship with tumor progression were analyzed via employing the GEPIA2 web portal (http://gepia2.cancer-pku.cn/) and TISIDB web portal (http://cis.hku.hk/TISIDB). Public HCC gene expression matrix was download from Gene Expression Omnibus (GEO) database, Liver Hepatocellular Carcinoma Project of The Cancer Genome Atlas (TCGA-LIHC), and Liver Cancer—RIKEN, JP Project from International Cancer Genome Consortium (ICGC-LIRI-JP). The correlation of *RCL1* expression with the abundance of immune infiltrates was obtained from the TIMER 2.0 (http://timer.cistrome.org/). The gene sets were downloaded from the Molecular Signatures Database (MSigDB) from the Gene Set Enrichment Analysis (GSEA) website (http://www.broadinstitute.org/gsea/msigdb/).

### Cell culture

All human HCC cell lines including Hep-3B, Huh-7, SNU-387, and Li-7, as well as normal human hepatocyte L-02, were obtained from the Cell Bank of the Chinese Academy of Sciences (Shanghai, China). Cells were maintained in Dulbecco’s Modified Eagle’s medium (DMEM; Hyclone, Logan, UT, USA), Minimum Essential Medium (MEM; Hyclone, Logan, UT, USA), or Roswell Park Memorial Institute-1640 (RPMI-1640, Hyclone, Logan, UT, USA) supplemented with 10% fetal bovine serum (FBS; LONSERA), streptomycin (100 mg/ml), and penicillin (100 unit/mL) at 37 ℃ in 5% CO_2_.

### Transient transfection for overexpression and knockdown of Rcl1

The overexpression vector targeting Rcl1 (FLAG-Rcl1) and a negative control (Ctrl) was conducted with assistance from Genechem CO., Ltd. (Shanghai, China). The Rcl1 short hairpin RNA (shRNA) vector (shRcl1) and its negative control (Ctrl) were also synthesized by Genechem CO., Ltd. (Shanghai, China). For Rcl1 knockdown, shRcl1 targeting the sequence of 5’GCATTGGTTTCTCCAACCTTA3’ and the control sequence 5’TTCTCCGAACGTGTACACGT3’. Transfections were performed using lipofectamine 2000 (Invitrogen) according to the manufacturer’s protocols.

### RNA extraction and quantitative real time-polymerase chain reaction (qRT-PCR)

Total RNA was extracted from HCC cell lines using EZ-press RNA Purification Kit (EZBioscience, China) and then reverse transcribed into cDNA using EZscript Reverse Transcription Mixture (EZBioscience, China) according to the manufacturer’s protocol. Quantitative Real-Time PCR was performed using the SYBR Green master mixture (EZBioscience, China) according to the manufacturer’s protocol. The following PCR primers were used:

Rcl1 forward: 5′-ATCTGTGGAACATGACTGTAGCG-3′;

Rcl1 reverse: 5′-ATCATTGGTCACTCCTCGTAGA-3′.

Tubulin forward: 5′-TGGACTCTGTTCGCTCAGGT-3′;

Tubulin reverse: 5′-TGCCTCCTTCCGTACCACAT-3′.

### Western blot (WB)

Cells were lysed in RIPA buffer (Beyotime Biotechnology, China) containing 1X Protease and Phosphatase inhibitor (Beyotime Biotechnology, China). An equal amount of protein samples was separated by 8% SDS/PAGE and transferred to 0.25 mm polyvinylidene fluoride membranes (Millpore, Germany). The membranes were blocked with 5% non-fat milk for 1 h, then incubated with the individual antibody at 4 ℃ overnight: Tubulin (YFB6011, YIFAN BIOLOGICAL), Rcl1 (15330-1-AP; Proteintech), GAPDH (bs-0755R, Bioss). The membranes were then incubated with the second antibody (bs-40295G-HRP, Bioss) at 37 ℃ for 1 h. Finally, protein bands were visualized using Omni ECL reagent (EpiZyme, China), and the gray intensity was acquired by using Fiji (NCBI, USA).

### Cell proliferation assay

Cell viability was monitored by Cell Count Kit 8 (Absin, China) according to the manufacturer’s protocol. Briefly, HCC cells were counted and plated onto 96-well cell culture plates at a density of 2 × 10^3^/well. Proliferation rates were measured by absorbance of 450 nm at 0, 1-, 2-, 3-, and 4-days post-transfection. Experiments were repeated more than three times with similar results.

### Immunofluorescence

HCC cells were seeded on a 35 × 35 mm confocal cell culture plate (Thermal, USA). After fixed using 4% paraformaldehyde, cultured cells were blocked with Immunol staining blocking buffer (Beyotime, China) with 0.3% Triton X-100 (Beyotine, China) in PBS for 30 min at RT. The samples were then incubated with primary antibody overnight at 4 ℃: Rcl1 (15330-1-AP; Proteintech), followed by the appropriate secondary fluorescently labeled antibodies (Invitrogen, USA) for 1 h at 37 ℃. Nuclei were counterstained with DAPI (Beyotime). Images were analyzed by a laser scanning confocal microscope (Olympus, FV300).

### Transwell assay

For migration assay, HCC cells were seeded (7 × 10^4^) onto the upper chamber wells with serum-free medium and then incubating for 48 h. The penetrated cells were fixed with 4% paraformaldehyde and stained with crystal violet. For the Matrigel invasion assay, the 24-well transwell chamber was coated with an extracellular matrix on the upper surface (Corning, USA). Then, HCC cells were seeded (1.5 × 10^5^) onto the upper chamber wells with serum-free medium. After incubating for 48 h, the penetrated cells were fixed with 4% paraformaldehyde and stained with crystal violet. Then, migrating or invading cells were photographed at 100 × and counted in five random fields.

### Cell cycle analysis

The cell cycle was analyzed by Cell Cycle Assay Kit Plus (US EVERYBRIGHT INC, China) according to the protocol. Briefly, HCC cells were collected and washed twice in cold phosphate-buffered saline (PBS), and then fixed in 70% cold ethanol at −20 ℃ for 24 h. The samples were washed twice in cold staining buffer and resuspended in 1 mL PBS with 4ul RedNucleus I staining solution. After incubation for 20 min at RT in the dark, the cell cycle was evaluated by flow cytometry (BD bioscience, USA). The ModFit LT5.0 (Verify Software House, USA) was used to analyze the cell cycle.

### Statistical analysis

Statistical analyses were performed using R software (version 4.0.0, USA) or GraphPad Prism (La Jolla, USA). The limma package was used to evaluate *Rcl1* expression between HCC tumor tissues and adjacent tissues using R software. The survival data of HCC patients were analyzed via log-rank test and Cox proportional hazard regression. According to the relevance of *Rcl1* expression, genes in TCGA-LIHC were divided into Rcl1^positive^ and Rcl1^negative^ subgroups. Gene set enrichment analysis (GSEA) was performed using R software. Continuous data were expressed as the mean ± standard deviation (SD). Comparisons between groups were performed using Student’s *t* test or ANOVA test. A value of *p* < 0.05 was defined as statistically significant.

## Results

### *RCL1* is abnormally expressed in various tumor tissues, and associated with prognosis and tumor progression

**Fig. 1:**
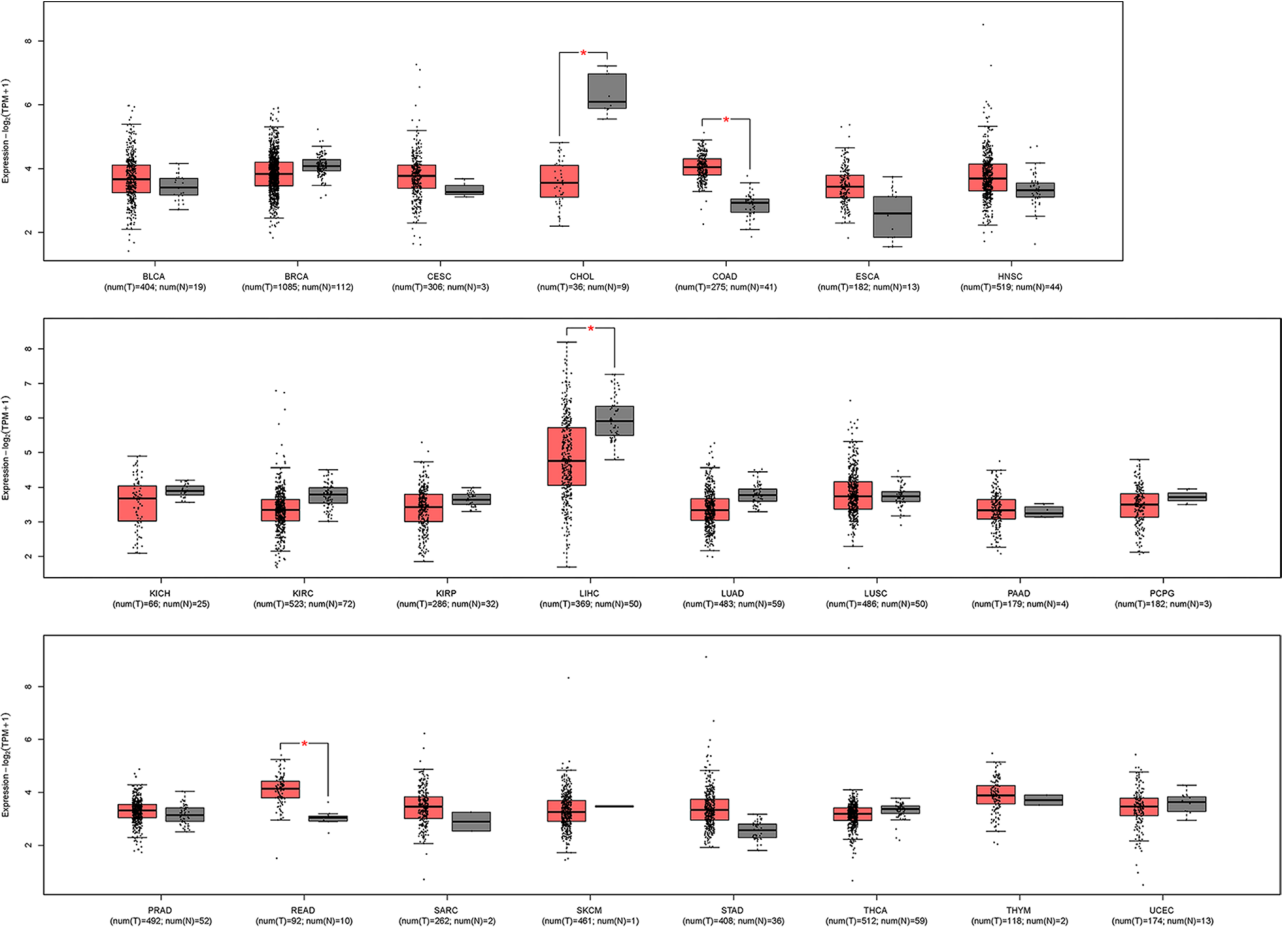
Human *RCL1* expression levels in different tumors and adjacent normal tissues from TCGA database. p-value Significant Codes: * < 0.05

We first explored the general expression of *Rcl1* in multiple human cancers using the GEPIA2 website portal (Fig. 1). The analyses of the RNA-seq data of 23 malignancies in TCGA suggested that the expression of *RCL1* was significantly lower in Cholangiocarcinoma and LIHC compared to the adjacent normal tissues. However, *RCL1* expression of Colon and Rectum adenocarcinomas were significantly higher than the one in normal tissues.

The TISIDB web portal was used (Additional file 1: Fig. S1A) to further evaluate the correlation of *RCL1* expression and survival prognosis. Notably, *RCL1* expression had a significant impact in the prognosis of 8 cancers, including brain lower-grade glioma (LGG), glioblastoma multiforme, kidney renal clear cell carcinoma (KIRC), LIHC, ovarian serous cystadenocarcinoma (OV), uterine corpus endometrial carcinoma (UCEC), uterine carcinosarcoma (UCS), and uveal melanoma (UVM). Low *RCL1* expression was remarkably associated with poor prognosis in all these cancer types except for UCEC and UCS.

Moreover, the association between *RCL1* expression and tumor progression across human cancers was identified. It was revealed that *RCL1* expression was positively correlated to tumor stage in KIRC, LIHC, and Stomach adenocarcinoma, as well as in UVM (Additional file 1: Fig. S1B). Similarly, the expression of *RCL1* in KIRC and LIHC was also positively associated with histological grade (Additional file 1: Fig. S1C). However, the *RCL1* downregulation was notably correlated with higher grade of Cervical squamous cell carcinoma and endocervical adenocarcinoma and UCEC (Additional file 1: Fig. S1C).

In summary, these results confirmed that RCL1 could be a potential tumor-associated gene in several malignancies. Notably, it was in LIHC that *RCL1* expression was not only significantly down-regulated but also associated with prognosis, tumor progression across many human cancers.

### Low *RCL1* expression is correlated with poor clinicopathological outcomes in HCC patients

**Fig. 2:**
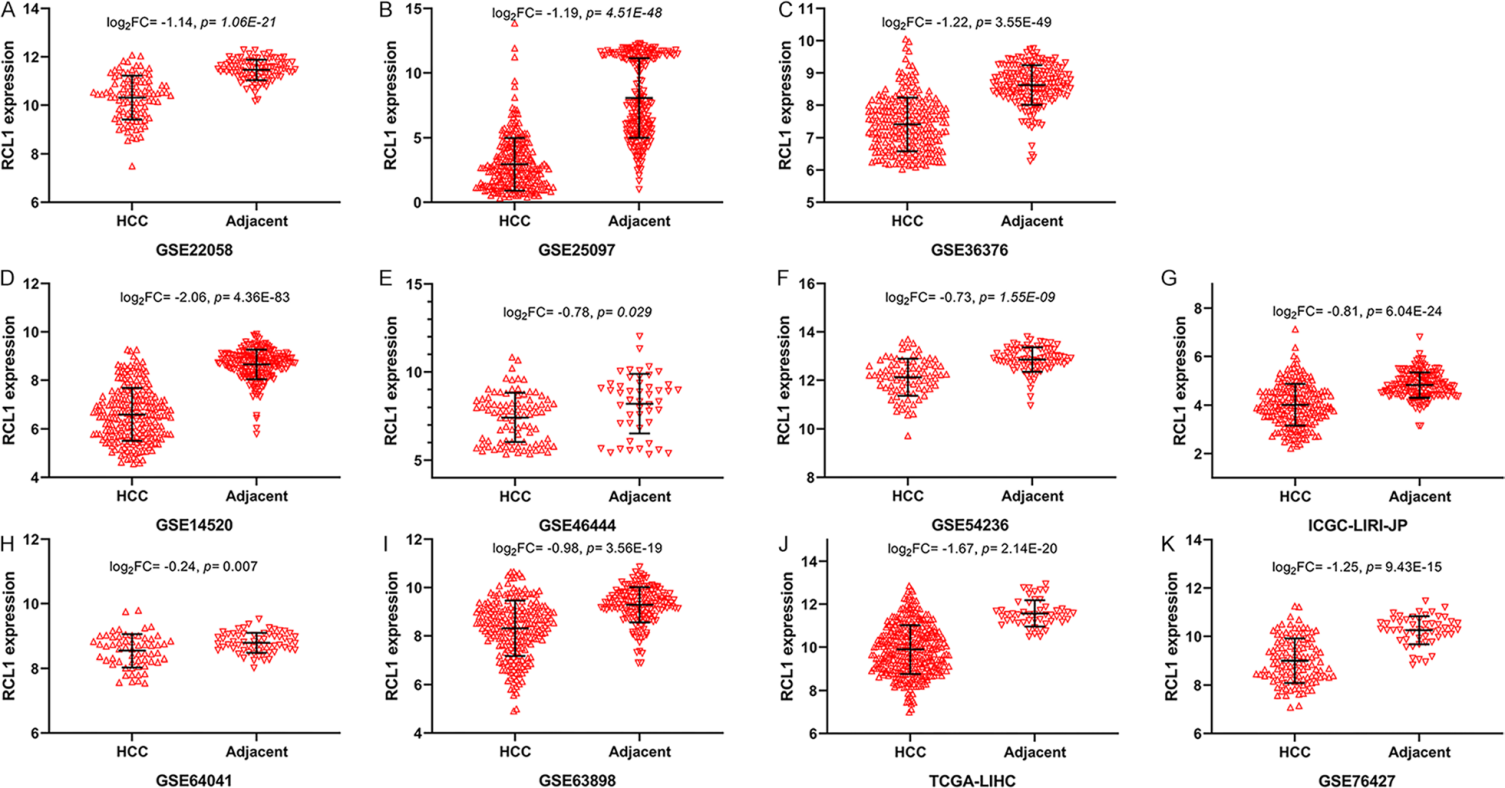
Expression of Rcl1 in patients with HCC based on data from TCGA-LIHC and GEO datasets. **A** GSE22058, **B** GSE25097, **C** GSE36376, **D** GSE14250 **E** GSE46444, **F** GSE54236, **G** ICGC-LIRI-JP, **H** GSE64041, **I** GSE63898, **J** TCGA-LIHC **K** GSE76427

Eleven HCC datasets were downloaded and analyzed to further verify *RCL1* expression in HCC tumor tissue. For 9 HCC datasets including GSE22058 (Fig. 2A), GSE25097 (Fig. 2B), GSE36376 (Fig. 2C), GSE14520 (Fig. 2D), GSE54236 (Fig. 2F), ICGC-LIR-JP (Fig. 2G), GSE63898 (Fig. 2I), TCGA-LIHC (Fig. 2J), and GSE76427 (Fig. 2K), the expression levels of *Rcl1* in HCC tissues were generally lower than the ones in adjacent tissues (*p* < 0.001).

**Fig. 3:**
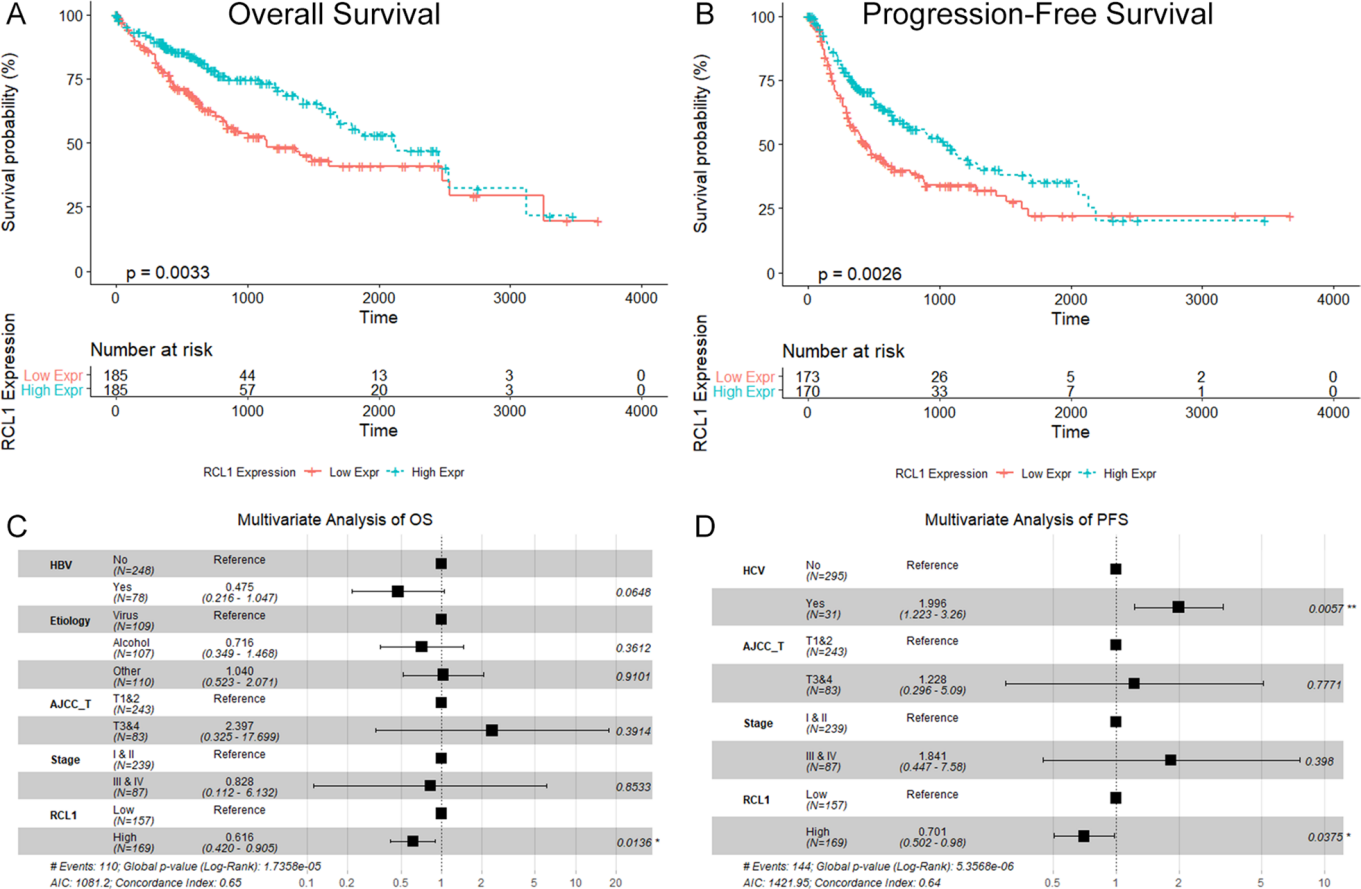
Patients with higher Rcl1 mRNA expression level had better prognosis in the TCGA-LIHC dataset. **A** Kaplan–Meier curve analysis for OS. **B** Kaplan–Meier analysis for PFS. **C** Forest plot of multivariate analysis for OS. **D** Forest plot of multivariate analysis for PFS. *OS* overall survival, *PFS* progression-free survival, *AJCC* American Joint Committee on Cancer

Univariate and multiple survival analyses were performed using R programming environment on the TCGA-LIHC dataset to promote understanding of the association between the *RCL1* expression and the prognosis of HCC. Univariate analysis indicated that patients with the high *RCL1* expression was associated with better overall survival (OS, HR = 0.607 (0.416–0.886)) and progression-free survival (PFS, HR = 0.661 (0.476–0.917)) (Fig. 3A, B). Univariate and multivariate analyses further confirmed that *RCL1* expression is an independent factor for OS (HR = 0.616 (0.420–0.905)) and PFS (HR = 0.701 (0.502–0.98)) of HCC patients (Fig. 3C, D, Table 1).

**Fig. 4:**
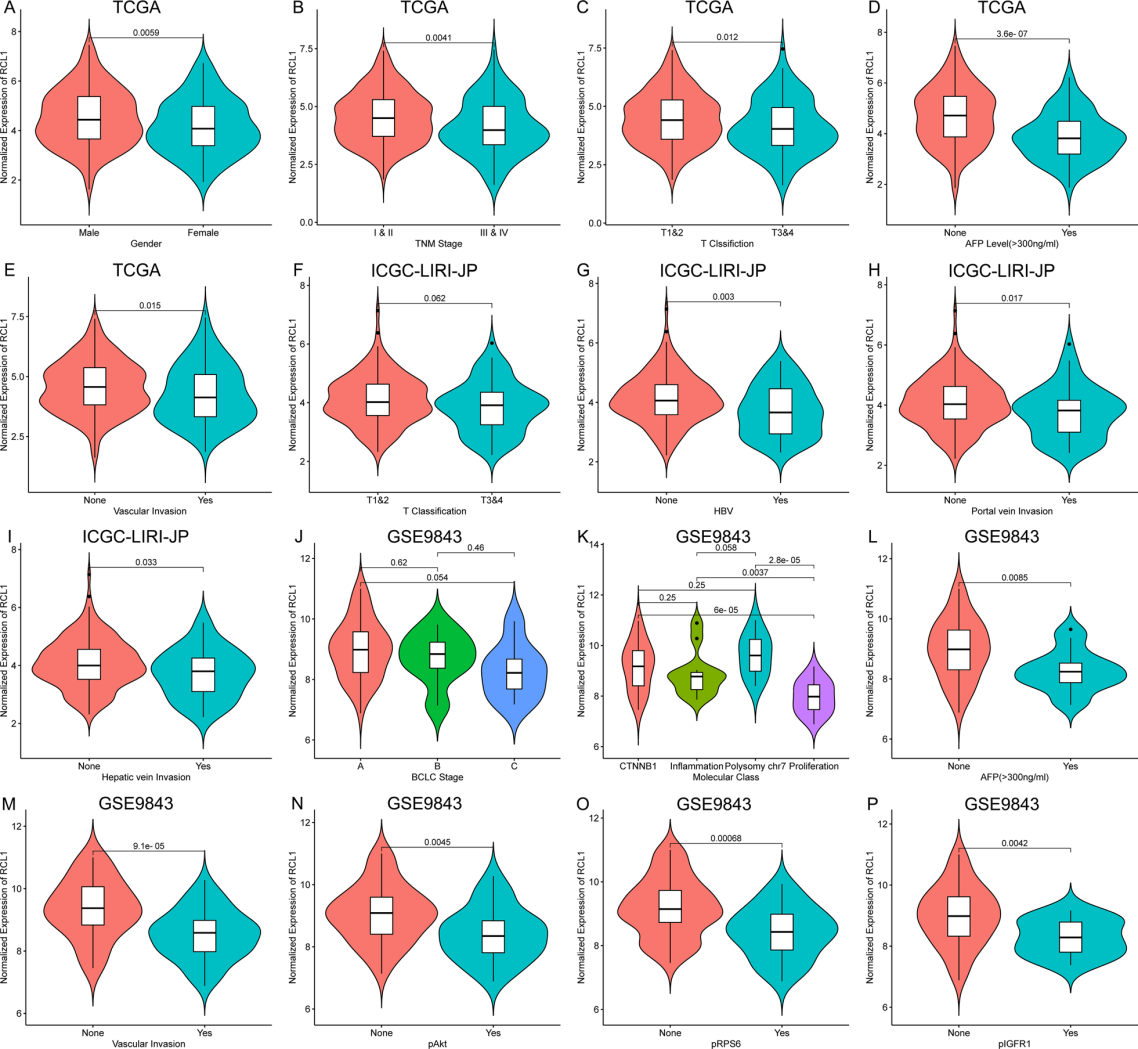
Association between *RCL1* expression and clinicopathologic characteristics in the HCC cohorts. *RCL1* expression was lower in **A** female, **B** higher TNM stage, **C** higher T classification, **D** higher AFP levels, **E** vascular invasion in TCGA cohort, **F** higher T classification, **G** HBV, **H** portal vein invasion, **I** hepatic vein invasion in ICGC cohort, and **J** BCLC C stage, **K** proliferation class, **L** high AFP level, **M** vascular invasion, and the positive expression of **N** phosphorylated Akt, **O** phosphorylated RPS6, **P** phosphorylated IGFR1 in GSE9843 cohort

In addition, we studied the connection between *RCL1* expression and the clinicopathological characteristics. It was found that low *RCL1* expression was significantly correlated with female, advanced primary tumor (T classification) and TNM stage, higher AFP level, as well as vascular invasion in TCGA-LIHC cohort (Fig. 4A–E). Besides, it was found that a decrease in the *RCL1* expression was associated with increasing T classification, HBV infection, portal vein and hepatic vein invasion in ICJC-LIRI-JP cohort (Fig. 4F–I). Meanwhile, a remarkable connection between *RCL1* expression was lower in the patients with BCLC C stage, proliferation class, high AFP level, vascular invasion, as well as phosphorylation level of Akt, RPS6, and IGFR1 in GSE9843 cohort (Fig. 4J–P). No significant relationship between the RCL1 mRNA expression and age (Additional file 2: Fig. S2A, C, F), cirrhosis (Additional file 2: Fig. S2B, E), as well as gender (Additional file 2: Fig. S2D, G) was found in HCC cohorts.

### *RCL1* expression significantly correlates with infiltrating levels of various immune cells in HCC

**Fig. 5:**
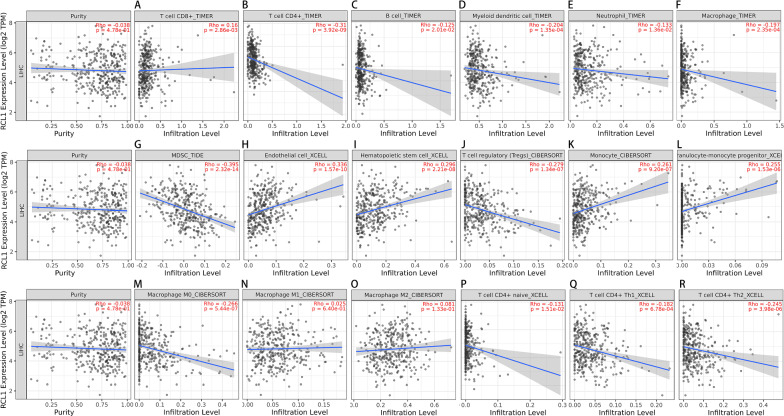
Correlation analyses between *RCL1* expression and immune cell infiltration in TCGA-LIHC cohort. **A** CD8^+^ T cell, **B** CD4^+^ T cell, **C** B cell, **D** Myeloid dendritic cell, **E** Neutrophil, **F** Macrophage, **G** myeloid-derived suppressor cell (MDSC), **H** Endothelial cell, **I** Hematopoietic stem cell, **J** T cell regulatory (Tregs), **K** Monocyte, **L** Granulocyte-monocyte progenitor, **M** CD4^+^ Th1 T cell, **N** CD4^+^ Th2 T cell, **O** CD4^+^ naïve T cell, **P** Macrophage M0, **Q** Macrophage M1, **R** Macrophage M2

The correlation of *RCL1* expression in HCC samples with immune infiltration levels were investigated using TIMER2.0 website portal. The results showed that *RCL1* expression significantly correlated with the infiltrating levels of myeloid-derived suppressor cell (MDSC, r = − 0.395, *p* = 2.32e−14, Fig. 5G), endothelial cell (r = 0.336, *p* = 1.57e−10, Fig. 5H), hematopoietic stem cell (r = 0.296, *p* = 2.21e−08, Fig. 5I), Tregs (r = − 0.279, *p* = 1.34e−07, Fig. 5J), monocyte (r = 0.261, *p* = 9.2e−07, Fig. 5K), granulocyte-monocyte progenitor (r = 0.255, *p* = 1.53e−06, Fig. 5L), CD4 + T cells (r = − 0.31, *p* = 3.92e−09, Fig. 5B), myeloid dendritic cell (r = − 0.204, *p* = 1.35e−04, Fig. 5D), macrophages (r = − 0.197, *p* = 2.35e−04, Fig. 5F), CD8 + T cells (r = 0.16, *p* = 2.81e−03, Fig. 5A), B cells (r = − 0.125, *p* = 2.01e−02, Fig. 5C), and neutrophils (r = − 0.133, *p* = 1.36e−02, Fig. 5E) in LIHC, although no significant correlation with tumor purity was found. Furthermore, the *RCL1* expression was negatively associated with the infiltration level of naïve CD4 + T cell (r = − 0.131, *p* = 1.51e−02, Fig. 5P), Th1 cell (r = − 0.182, *p* = 6.78e−04, Fig. 5Q), Th2 cell (r = − 0.245, *p* = 3.98e−06, Fig. 5R), M0 macrophages (r = − 0.266, *p* = 5.44e−07, Fig. 5M), but not with M1 macrophages (r = 0.025, *p* = 6.40e−01, Fig. 5N) and M2 macrophages (r = − 0.081, *p* = 1.33e−01, Fig. 5O).

**Fig. 6:**
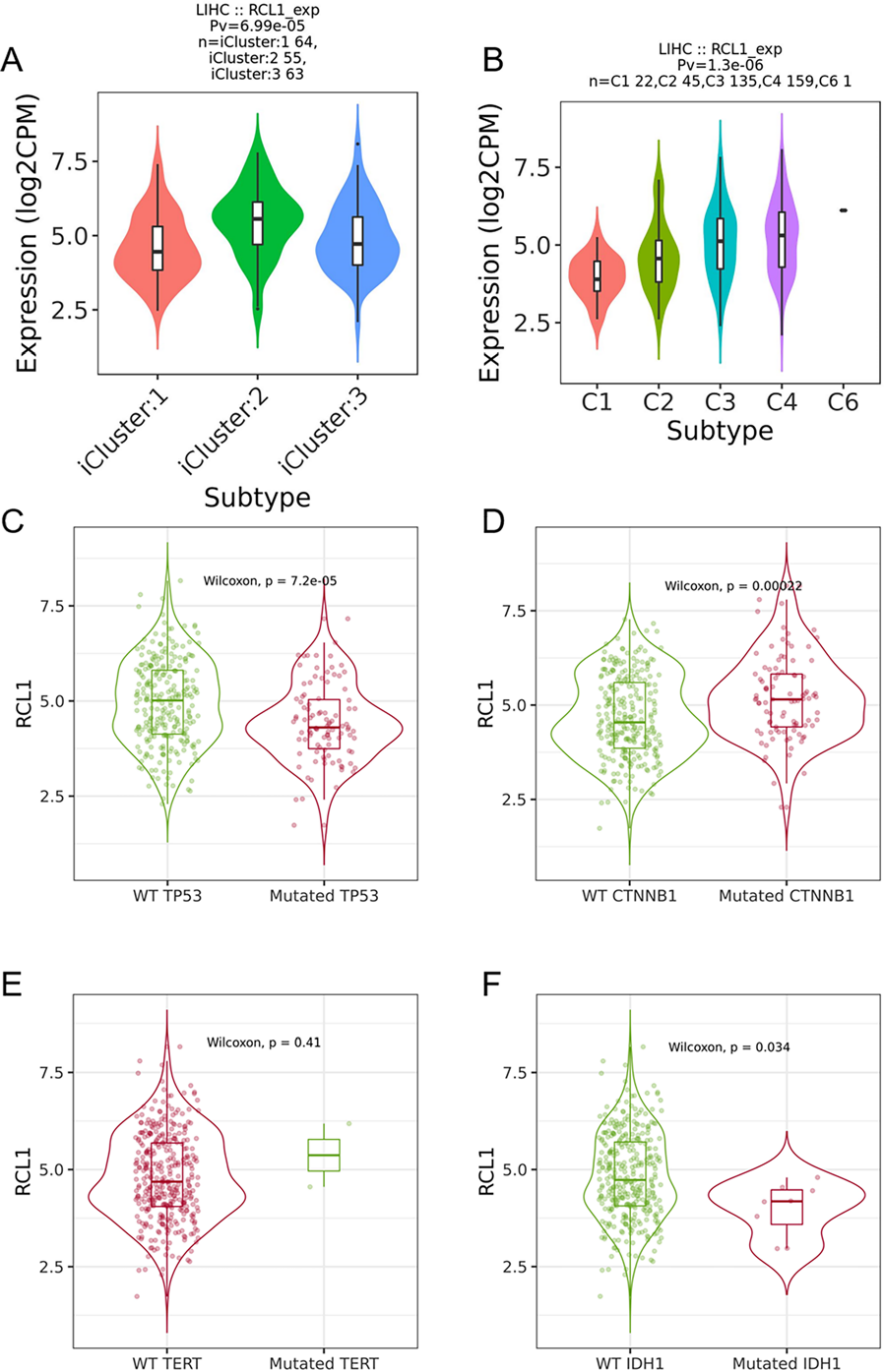
*RCL1* expression is strongly correlated with molecular- and immune-classifications of HCC. **A** Violin Plot of the *RCL1* expression in different molecular subtypes. **B** Violin Plot of the *RCL1* expression in different immune subtypes. C1 Wound healing, C2 IFN-γ Dominant, C3 inflammatory, C4 Lymphocyte depleted, C6 TGF-β Dominant. **C** Violin Plot visualizing the *RCL1* expression in wild-type and mutant TP53 gene. **D** Violin Plot visualizing the *RCL1* expression in wild-type and mutant CTNNB1 gene. **E** Violin Plot visualizing the *RCL1* expression in wild-type and mutant TERT gene. **F** Violin Plot visualizing the *RCL1* expression in wild-type and mutant IDH1 gene

Moreover, *RCL1* expression was found to be significantly different between molecular and immune subtypes by exploring the TISIDB web portal (p < 0.001, Fig. 6A, B). Furthermore, the expression levels of *RCL1* in the patients with TP53 and IDH1 mutation were lower than the ones in the patients with TP53 and IDH1 wild-type (Fig. 6C, D). The expression levels of *RCL1* in the patients with CTNNB1 mutation were higher than the ones in the patients with CTNNB1 wild-type (Fig. 6E) and no significant difference was observed between the expression levels of *RCL1* in TERT mutation and wild-type patients (Fig. 6F).

### Rcl1 is down expression in HCC cells and suppresses HCC cell growth and metastasis in vitro

**Fig. 7:**
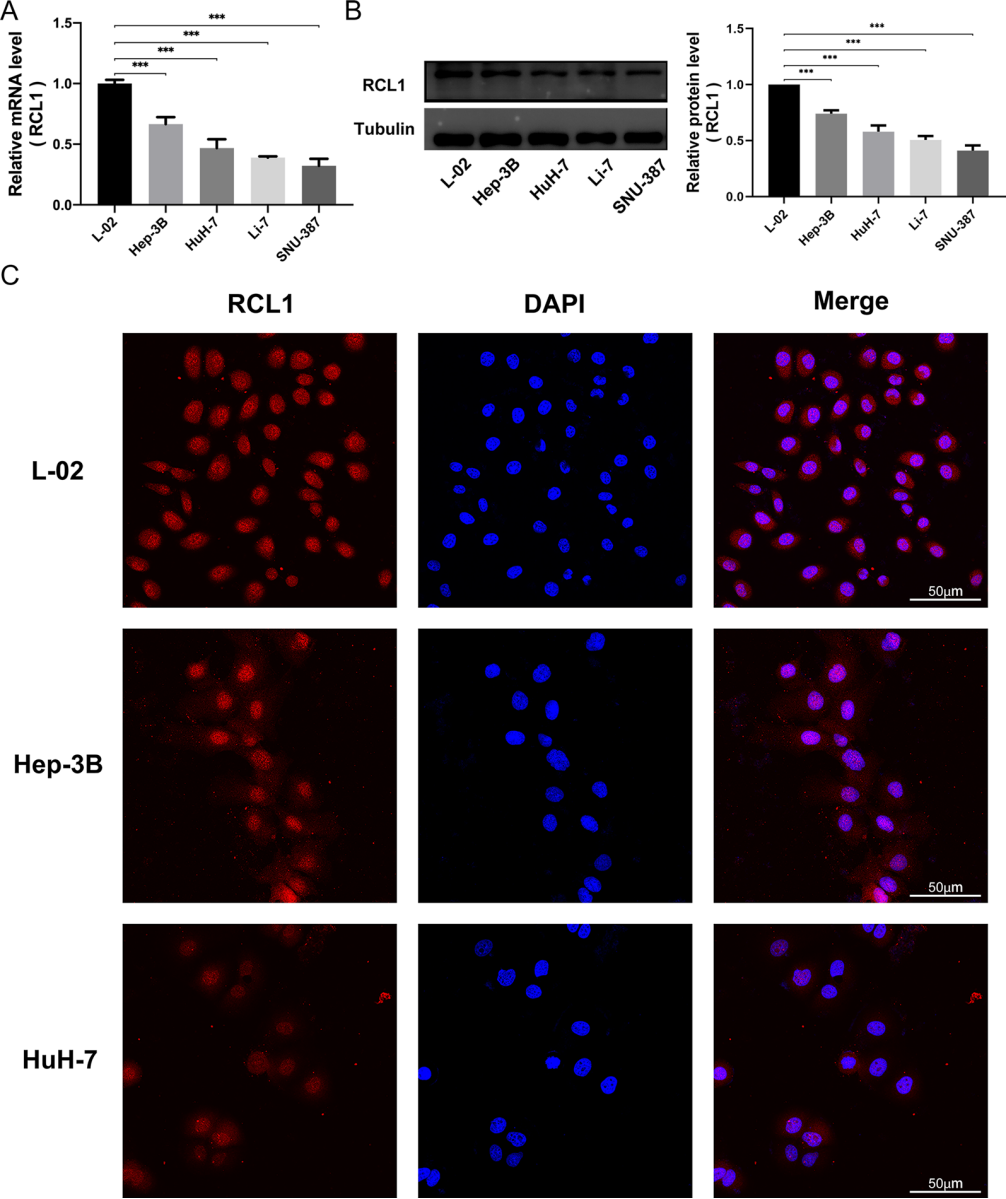
The Rcl1 expression in HCC cell lines. **A** Analysis of the Rcl1 mRNA expression in HCC cell lines. **B** Western blot analysis of the Rcl1 protein expression in HCC cell lines. **C** Immunofluorescence staining of the Rcl1 protein in HCC cell lines. p-value significance symbols: * < 0.05, ** < 0.01, *** < 0.001. Bar = 50 μm

The endogenous Rcl1 expression levels were detected in a collection of liver cancer cell lines and L-02 cells. Both mRNA and protein expression levels of Rcl1 were generally lower in all liver cancer lines in comparison with L-02 cells (Fig. 7A, B). The Rcl1 expression of high-invasive HCC cell lines were substantially lower than the one in the low-invasive cell lines. These findings were further supported by immunofluorescence staining. Interestingly, it was also revealed that there was a marked difference in the distribution of Rcl1 protein between the liver cells and cancer cells. In particular, Rcl1 was mostly located in the nucleus in the HCC cell lines, while it was uniformly distributed in nucleus and cytoplasm in the liver cell lines (Fig. 7C).

**Fig. 8:**
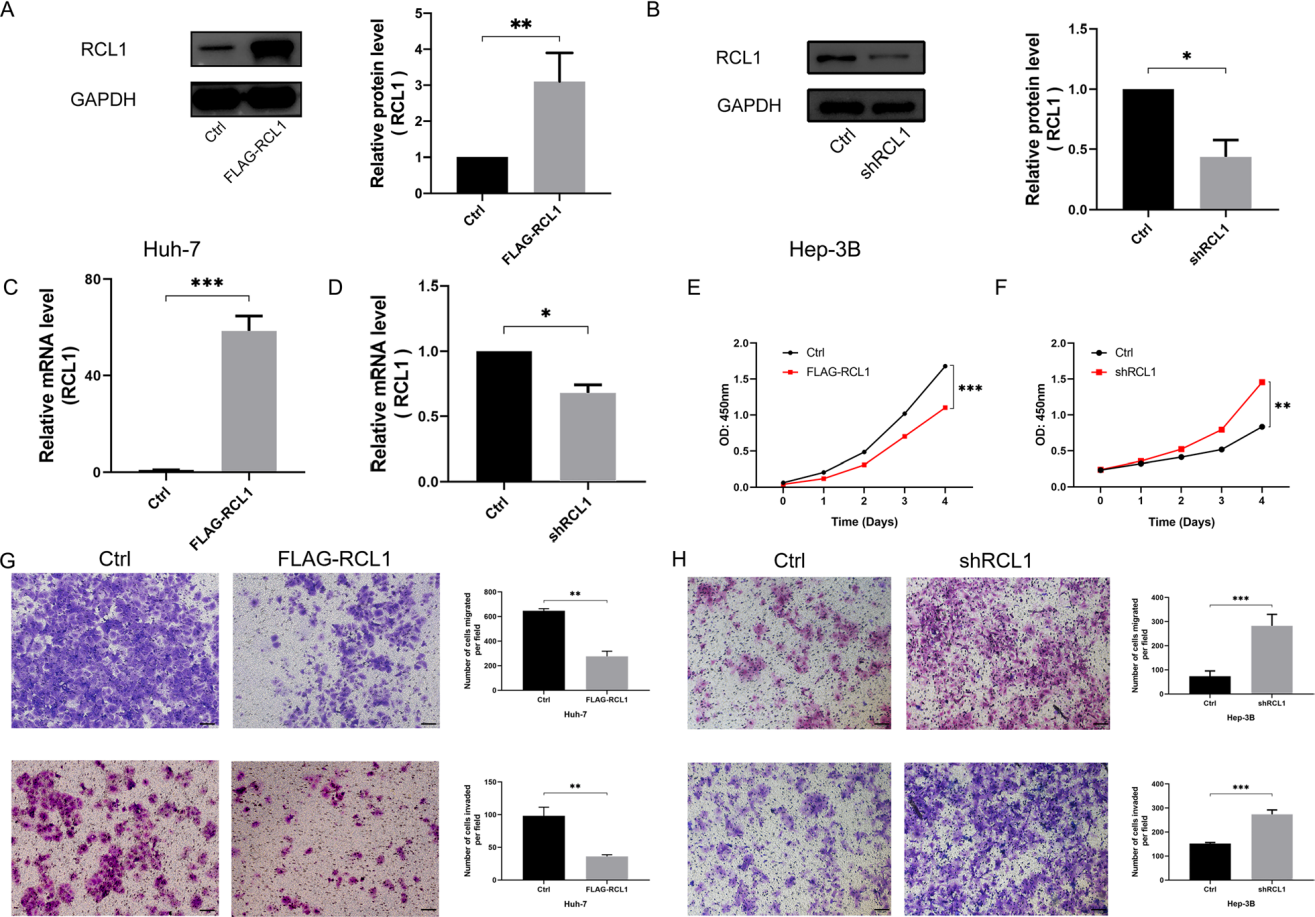
Rcl1 inhibits HCC cells proliferation, migration and invasion in vitro. **A**, **B** western blot analyses and **C**, **D** qRT-PCR assays of Rcl1 overexpression and knockdown in Huh-7 and Hep-3B cells. **E**, **F** CCK-8 assays of Huh-7 and Hep-3B cells after Rcl1 overexpression or knockdown with the corresponding negative controls. **G**, **H** Transwell assays examined the migration and invasion activities of Rcl1 overexpression or knockdown with their corresponding negative controls. Data are presented as the mean ± standard deviation from three independent experiments. *p < 0.05; **p < 0.01; ***p < 0.001. Bar = 100 μm

Then a recombinant plasmid vector encoding Rcl1 (FLAG-Rcl1) was conducted and an empty vector was used as control (Ctrl). The overexpression of Rcl1 in Huh-7 cells was validated with RT-PCR and western blot analyses (Fig. 8A, C). Forced Rcl1 expression could markedly inhibit cell growth as supported by cell viability assay in Huh-7 cell (Fig. 8E). Moreover, transwell assays indicated that the overexpression of Rcl1 significantly impaired Huh-7 cell’s ability to migrate and invade (Fig. 8G). Furthermore, Rcl1 was knockdown by transfecting the shRcl1 vector or empty vector into Hep-3B cell line. The efficiency of knockdown was confirmed by RT-PCR and western blot analyses (Fig. 8B, D). Consistently, knockdown of Rcl1 in Hep-3B cell strikingly enhanced cell viability, migration, and invasion (Fig. 8F, H).

### Rcl1 could potentially participate in regulating cell cycle and metabolism-associated signal pathways

**Fig. 9:**
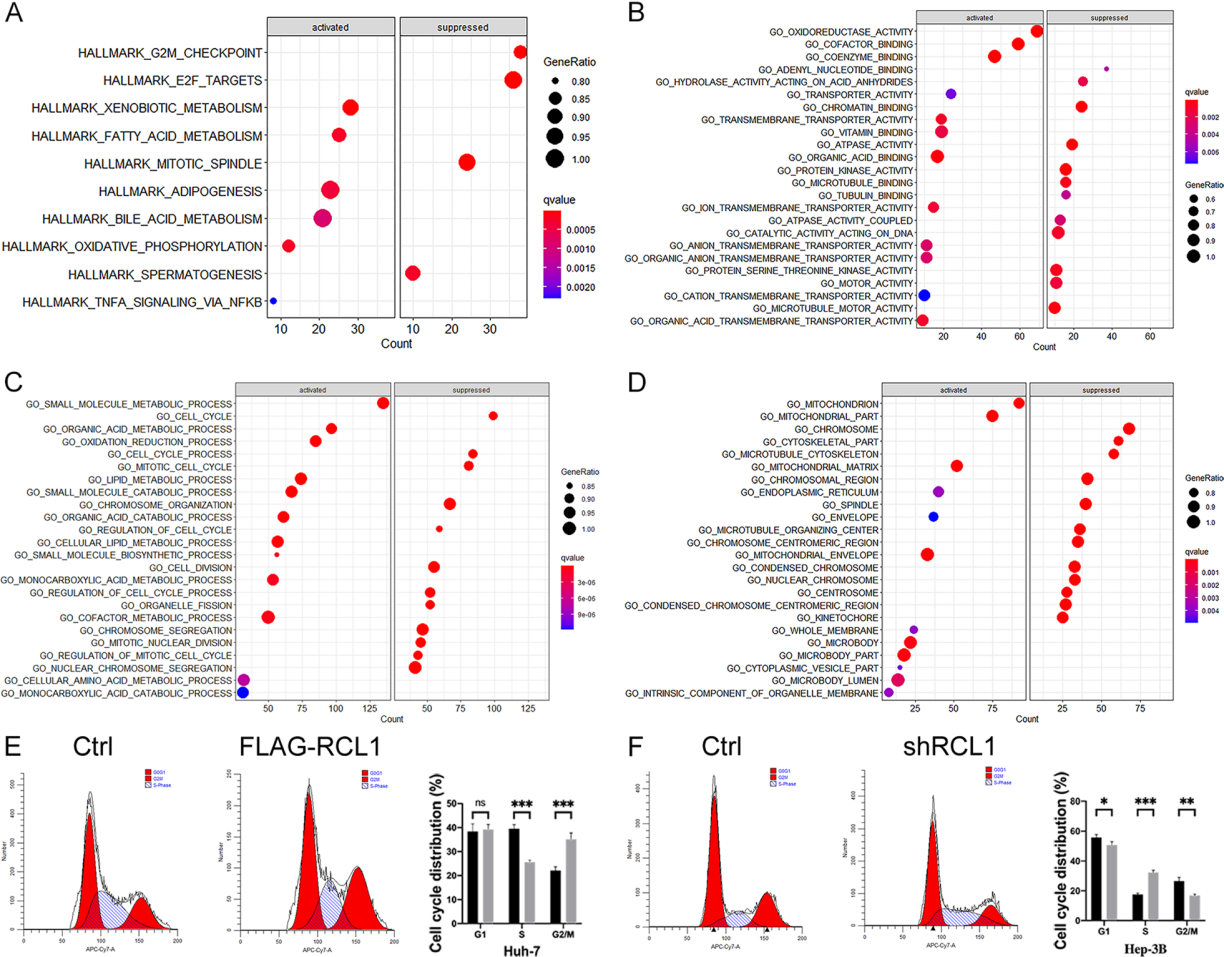
Rcl1 is strongly correlated with cell cycle- and metabolism-related pathways. **A** Scatter plot of the significantly enriched Hallmark gene sets. Top 10 enriched pathways are shown in **B**, **C**, **D** The significantly enriched Gene Ontology annotations (molecular functions, biological processes, and cellular components). Top 20 enriched pathways are shown in **E**, **F**) Cell cycle tests of Huh-7 and Hep-3B cells after Rcl1 overexpression or knockdown with their corresponding controls. NS > 0.05; *p < 0.05; **p < 0.01; ***p < 0.001

Notably, the Rcl1^positive^ group of genes were enriched in multiple cellular metabolic processes such as xenobiotic, fatty acid, bile acid, adipogenesis, and oxidative phosphorylation, while the Rcl1^negative^ subgroup of genes were enriched in cell cycle regulation including G2M checkpoint, E2F targets and mitotic spindle (Fig. 9A, C). Moreover, gene ontology (GO) analysis revealed that Rcl1 could potentially promote the activation of protein-binding and transmembrane transport, while simultaneously inhibiting microtubule and protein kinase activity (Fig. 9B). Mitochondria and chromosomes were the main cellular organelles of the Rcl1^positive^ and Rcl1^negative^ groups, respectively (Fig. 9D).

The effects of Rcl1 on cell cycle distribution was evaluated using flow cytometry analysis. We found that the overexpression of Rcl1 resulted in a significant increase of cells in G2M phase (35.10% vs 22.06%, *p* = 0.002) and a concomitantly significant decrease of cells in S phase (25.62% vs 39.53%, *p* = 0.0002) in Huh-7 cell (Fig. 9E). As expected, upon Rcl1 knockdown, the ratio of cells in G2M phase was decreased (18.89% vs 26.58%, *p* = 0.003), the ratio in S phase was increased (32.33% vs 17.59%, *p* = 0.0001), and the ratio in the G0/1 phase was decreased (50.78% vs 55.84%, *p* = 0.040) (Fig. 9F).

## Discussion

Rcl1 could interact with Bms1 to endonucleolytic cleave the pre-rRNA at the A2 site, which is required for the eukaryotic pre-ribosome assembly implicated in the 18 s rRNA biogenesis [17–19]. However, the mechanism that allows Rcl1 to carry out the catalytic activity in human species still remains unclear since the mutation of the proposed pre-rRNA substrate binding site of human Rcl1 does not affect 2a cleavage or 18S production [15]. In the present study, we found that the expression levels of *RCL1* was primarily reduced in HCC tissues and cell lines. Moreover, its expression significantly correlated with the survival prognosis, tumor progression, immune infiltration level, as well as molecular classification of HCC. Furthermore, both gain- and loss-of-function experiments demonstrated that Rcl1 has the inhibitory effects on the proliferation, migration, and invasion of HCC cells in vitro. Bioinformatic analyses revealed that *Rcl1* could be significantly associated with cell cycle transition, cellular metabolic processes. Mechanistically, it was identified that Rcl1 overexpression or knockdown could inhibit or promote cell cycle transition.

*RCL1* was found to be up-regulated in colorectal cancers, but downregulated in liver and bile duct cancers. Venkateswaran et al. found that non-coding variants of *RCL1* could regulate granulocyte–macrophage colony-stimulating factor signaling in inflammatory bowel disease patients [20]. It is likely that single nucleotide polymorphism of *RCL1* influences the gut microbiome. Gut microbiome has a key role in carcinogenesis and anticancer immune response of HCC and colorectal cancer [21, 22]. And low *RCL1* expression was associated with poor prognosis and tumor stage in multiple cancers. It suggested that Rcl1 may play a dualistic role by acting both as an oncogene and tumor suppressor, which is similar to other ribosome biogenesis factors [23].

Many studies have elucidated that many ribosome assembly factors could promote cell growth and metastasis in HCC by up-regulating the rate of ribosome biogenesis [9, 24–28]. But we found that *RCL1* expression was commonly downregulated in HCC tissues and cell lines. Besides, bioinformatic analyses indicated that low *RCL1* expression was the risk factor of poor survival prognosis and tumor progression, including: advanced TNM classification, high AFP level, vascular invasion in many HCC cohorts. And *RCL1* expression was relatively lowest in iCluster 1 and proliferation molecular subtypes of HCC which exhibited the high frequency of macrovascular invasion and a significantly worse prognosis [29]. Recently, Zhu et al. reported that Rcl1 is essential for the 18S rRNA maturation at A1-site and for digestive organogenesis in zebrafish [30]. And its deficiency may upregulate the expression of genes responsible for ribosome biogenesis. Meanwhile, we also found that Rcl1 protein was distributed in both nucleus and cytoplasm of HCC cell and hepatocyte lines by immunofluorescence, which is consistent with a recent study [31]. Notably, the cytoplasmic Rcl1 protein level of HCC cell lines was dramatically reduced compared to hepatocyte cell. And it has been documented that the localization of BOP1, one of ribosome biogenesis factors, from nucleus to cytoplasm correlated with advanced disease and decreased survival in prostate cancer patients. Further research is needed to be proven whether Rcl1 could participate in the biological processes in the cytoplasm.

Moreover, functional tests indicated Rcl1 may be a potential tumor suppressor in HCC in vitro. Mechanistically, GSEA also suggested RCL1 was involved in cell cycle control and multiple cellular metabolic processes. It was further identified that Rcl1 overexpression could induce HCC cell cycle arrest by flow cytometer analysis. Consistently, Rcl1 knockdown obviously promote cell cycle progression of HCC cell. Large number of studies have demonstrated that aberrant pre-rRNA intermediates could lead to cell cycle arrest [32–35]. The most widely accepted model of cell-cycle arrest, upon pre-rRNA processing defect, was that the PeBoW complex could increase the extra-ribosome RPL5-RPL11-5S rRNA complex to bind and block the human homolog of mouse double minute-mediated ubiquitination and degradation of p53 [34, 36–38]. But it was documented that exogenous expression of Rcl1 in senescent cells could not increase the accumulation of p53, p21, and p16 [39]. Combining the bioinformatic analyses and flow cytometer analysis, we found that Rcl1 mainly control cell cycle progression by interfering the S phase.

Immune-related mechanisms play notable roles in the incidence and recurrence of HCC, and a combination of molecular and immune therapies could remarkably increase objective response in advanced HCC by 30–40% [40]. Ribosome biogenesis modulate immunosurveillance and innate immune response [41–43]. We also examined the correlation between the RCL1 expression and the infiltration levels of several immune cells in the TCGA-LIHC cohort. There exists a moderate negative relationship between the *Rcl1* expression and the infiltration level of MDSC, CD4 + T cell, and Tregs, and significantly positive correlations with the infiltration level of endothelial cell, hematopoietic stem cell, and CD8 + T cells. MDSC contributes to the immunosuppressive network through multiple mechanisms and mediates the tumor growth, angiogenesis, and metastasis of HCC [44] while endothelial cell necroptosis induced by tumor-cells could reversely promote cancer metastasis [45]. Moreover, we also found a prominent association between the expression levels of *RCL1* and the immune-subtypes in HCC patients. The *RCL1* expression was relatively lower in the C1 subtype (wound healing). Its characteristics include a high proliferation rate, the upregulated expression of angiogenic genes, a Th2 cell-dominated immune infiltration, as well as less favorable outcomes. Recently, Jung et al. found that an rRNA fragment containing 2’,3’-cyclic phosphate and guanosine triphosphate (GTP) -binding activity functions as an endogenous RIG-I ligand to induce immune stimulation [46]. It is generally accepted that the interaction between Rcl1 and Bms1, a GTPase-activating protein, is involved in pre-rRNA processing across species [17, 18]. Further observational studies are required to confirm whether the Rcl1-Bms1 complex could module the innate stimulation by activating RIG-I.

Our study has several limitations. First, this was a retrospective analysis based on public databases (TCGA and GEO). Although Rcl1 expression in human HCC cell lines has been analyzed, it should still be determined in clinical specimens. Second, despite in vitro experiments suggested that Rcl1 may be a tumor suppressor in HCC, additional animal tests should be performed. In addition, the exact molecular mechanism regarding Rcl1on cell cycle regulation should be further explored.

## Conclusions

In summary, our study demonstrates that Rcl1 could serve as a favorable prognostic factor for HCC. Moreover, intracellular molecular metabolism and cell cycle control might be the primary biological processes regulated by Rcl1. Furthermore, the results of cell experiment indicated that Rcl1 plays a pivotal anti-cancer role by inhibiting of both growth and metastasis of HCC. The significant reduction of cytoplasmic Rcl1 protein in HCC imply the additional biological function. Our study revealed that Rcl1 may act as a potential prognostic marker and tumor suppressor in HCC.

**Table 1:** Univariate analysis of TCGA-LIHC patient overall survival and progression-free survival

Parameters	Overall survival			Progression-free survival		
	HR	95%CI	*P* value	HR	95%CI	*P* value
Age	1.070	0.717–1.597	0.741	0.909	0.645–1.281	0.585
Gender	0.750	0.511–1.100	0.14	0.957	0.674–1.36	0.808
HBV	0.470	0.280–0.791	**0.004**	0.734	0.498–1.082	0.118
HCV	1.134	0.590–2.179	0.707	1.703	1.048–2.766	**0.032**
Etiology	1.442	0.693–1.821	**0.002**	1.035	0.846–1.265	0.74
AJCC_T	2.369	1.617–3.471	**0.000**	2.242	1.582–3.178	**0.000**
Stage	2.348	1.605–3.436	**0.000**	2.257	1.598–3.188	**0.000**
RCL1	0.607	0.416–0.886	**0.009**	0.661	0.476–0.917	**0.01**

TNM tumor-nodes-metastases

AJCC American Joint Committee on Cancer

Statistically significant *P* value (*p* < 0.05) were bold processed

## Supplementary Information

**Additional file 1: Fig. S1.** The landscape of relationship between Rcl1 mRNA expression and overall survival, tumor stage, histological grade in different types of cancer. (A) Overall survival (B) Tumor stage, (C) Histological grade. Longer (or Shorter): the gene is associated with longer (shorter) survival (Log rank test: *p* < 0.05); Lower (or Higher): the gene is associated with lower (or higher) stage (Spearman correlation test: *p* < 0.05); Lower (or Higher): the gene is associated with lower (or higher) stage (Spearman correlation: *p* < 0.05); NS No significant.

**Additional file 1: Fig. S2.** Association between *RCL1* expression and clinicopathologic characteristics in the HCC cohorts. No significant correlation between *RCL1* expression and (A) age (B) cirrhosis in TCGA cohort, (C) age (D) gender (E) cirrhosis in ICGC cohort, (F) age (G) gender in GSE9843 cohort.

## Data Availability

The RNA-sequencing data and corresponding clinical information were downloaded from The Cancer Genome Atlas (TCGA) database (https://portal.gdc.cancer.gov/), International Cancer Genome Consortium (ICGC) databases (https://dcc.icgc.org/), Gene Expression Omnibus (GEO) databases (https://www.ncbi.nlm.nih.gov/geo/).

## Abbreviations

DMEM: Dulbecco’s Modified Eagle’s medium
EMT: Epithelial mesenchymal transition
FBS: Fetal bovine serum
GO: Gene ontology
GSEA: Gene set enrichment analysis
GTP: Guanosine triphosphate
KIRC: Kidney renal clear cell carcinoma
LGG: Brain lower-grade glioma
LIHC: Liver hepatic carcinoma
MEM: Modified Eagle’s medium
OS: Overall survival
PFS: Progression free survival
qRT-PCR: Quantitative real time-polymerase chain reaction
Rcl1 RNA: 3’-Terminal phosphate cyclase-like protein
RPMI-1640: Roswell Park Memorial Institute-1640
UCES: Uterine corpus endometrial carcinoma
UCS: Uterine carcinosarcoma
UVM: Uveal melanoma
WB: Western blot

## References

[1] Sung H, Ferlay J, Siegel RL, Laversanne M, Soerjomataram I, Jemal A (2021). Global cancer statistics 2020: GLOBOCAN estimates of incidence and mortality worldwide for 36 cancers in 185 countries. CA Cancer J Clin.

[2] Llovet JM, Kelley RK, Villanueva A, Singal AG, Pikarsky E, Roayaie S, Lencioni R, Koike K, Zucman-Rossi J, Finn RS (2021). Hepatocellular carcinoma. Nat Rev Dis Primers.

[3] Ford D (2020). Ribosomal heterogeneity—a new inroad for pharmacological innovation. Biochem Pharmacol.

[4] Shi Z, Fujii K, Kovary KM, Genuth NR, Rost HL, Teruel MN, Barna M (2017). Heterogeneous ribosomes preferentially translate distinct subpools of mRNAs genome-wide. Mol Cell.

[5] Genuth NR, Barna M (2018). The discovery of ribosome heterogeneity and its implications for gene regulation and organismal life. Mol Cell.

[6] Bohnsack KE, Bohnsack MT (2019). Uncovering the assembly pathway of human ribosomes and its emerging links to disease. EMBO J.

[7] Pelletier J, Thomas G, Volarević S (2018). Ribosome biogenesis in cancer: new players and therapeutic avenues. Nat Rev Cancer.

[8] Chen ZH, Qi JJ, Wu QN, Lu JH, Liu ZX, Wang Y, Hu PS, Li T, Lin JF, Wu XY (2019). Eukaryotic initiation factor 4A2 promotes experimental metastasis and oxaliplatin resistance in colorectal cancer. J Exp Clin Cancer Res.

[9] Ruan Y, Sun L, Hao Y, Wang L, Xu J, Zhang W, Xie J, Guo L, Zhou L, Yun X (2012). Ribosomal RACK1 promotes chemoresistance and growth in human hepatocellular carcinoma. J Clin Investig.

[10] Kearse MG, Goldman DH, Choi J, Nwaezeapu C, Liang D, Green KM, Goldstrohm AC, Todd PK, Green R, Wilusz JE (2019). Ribosome queuing enables non-AUG translation to be resistant to multiple protein synthesis inhibitors. Genes Dev.

[11] Lawrence MG, Obinata D, Sandhu S, Selth LA, Wong SQ, Porter LH, Lister N, Pook D, Pezaro CJ, Goode DL (2018). Patient-derived models of abiraterone- and enzalutamide-resistant prostate cancer reveal sensitivity to ribosome-directed therapy. Eur Urol.

[12] Billy E, Wegierski T, Nasr F, Filipowicz W (2000). Rcl1p, the yeast protein similar to the RNA 3'-phosphate cyclase, associates with U3 snoRNP and is required for 18S rRNA biogenesis. EMBO J.

[13] Horn DM, Mason SL, Karbstein K (2011). Rcl1 protein, a novel nuclease for 18 S ribosomal RNA production. J Biol Chem.

[14] Tanaka N, Smith P, Shuman S (2011). Crystal structure of Rcl1, an essential component of the eukaryal pre-rRNA processosome implicated in 18s rRNA biogenesis. RNA.

[15] Wells GR, Weichmann F, Colvin D, Sloan KE, Kudla G, Tollervey D, Watkins NJ, Schneider C (2016). The PIN domain endonuclease Utp24 cleaves pre-ribosomal RNA at two coupled sites in yeast and humans. Nucleic Acids Res.

[16] Minguez B, Hoshida Y, Villanueva A, Toffanin S, Cabellos L, Thung S, Mandeli J, Sia D, April C, Fan JB (2011). Gene-expression signature of vascular invasion in hepatocellular carcinoma. J Hepatol.

[17] Delprato A, Al Kadri Y, Pérébaskine N, Monfoulet C, Henry Y, Henras AK, Fribourg S (2014). Crucial role of the Rcl1p-Bms1p interaction for yeast pre-ribosomal RNA processing. Nucleic Acids Res.

[18] Wang Y, Zhu Q, Huang L, Zhu Y, Chen J, Peng J, Lo LJ (2016). Interaction between Bms1 and Rcl1, two ribosome biogenesis factors, is evolutionally conserved in zebrafish and human. J Genet Genomics.

[19] Tafforeau L, Zorbas C, Langhendries J-L, Mullineux S-T, Stamatopoulou V, Mullier R, Wacheul L, Lafontaine Denis LJ (2013). The complexity of human ribosome biogenesis revealed by systematic nucleolar screening of pre-rRNA processing factors. Mol Cell.

[20] Venkateswaran S, Denson LA, Jurickova I, Dodd A, Zwick ME, Cutler DJ, Kugathasan S, Okou DT (2019). Neutrophil GM-CSF signaling in inflammatory bowel disease patients is influenced by non-coding genetic variants. Sci Rep.

[21] Song M, Chan AT, Sun J (2020). Influence of the gut microbiome, diet, and environment on risk of colorectal cancer. Gastroenterology.

[22] Schwabe RF, Greten TF (2020). Gut microbiome in HCC—mechanisms, diagnosis and therapy. J Hepatol.

[23] Srinivas AN, Suresh D, Mirshahi F, Santhekadur PK, Sanyal AJ, Kumar DP (2021). Emerging roles of AATF: checkpoint signaling and beyond. J Cell Physiol.

[24] Wang J, Sun J, Zhang N, Yang R, Li H, Zhang Y, Chen K, Kong D (2019). PES1 enhances proliferation and tumorigenesis in hepatocellular carcinoma via the PI3K/AKT pathway. Life Sci.

[25] Wang H, Xiao W, Zhou Q, Chen Y, Yang S, Sheng J, Yin Y, Fan J, Zhou J (2009). Bystin-like protein is upregulated in hepatocellular carcinoma and required for nucleologenesis in cancer cell proliferation. Cell Res.

[26] Yin Y, Zhou L, Zhan R, Zhang Q, Li M (2018). Identification of WDR12 as a novel oncogene involved in hepatocellular carcinoma propagation. Cancer Manag Res.

[27] Zhang X, Chen J, Jiang S, He S, Bai Y, Zhu L, Ma R, Liang X (2019). N-Acetyltransferase 10 enhances doxorubicin resistance in human hepatocellular carcinoma cell lines by promoting the epithelial-to-mesenchymal transition. Oxid Med Cell Longev.

[28] Chung K-Y, Cheng IKC, Ching AKK, Chu J-H, Lai PBS, Wong N (2011). Block of proliferation 1 (BOP1) plays an oncogenic role in hepatocellular carcinoma by promoting epithelial-to-mesenchymal transition. Hepatology.

[29] Cancer Genome Atlas Research Network Electronic address wbe, Cancer Genome Atlas Research N (2017). Comprehensive and integrative genomic characterization of hepatocellular carcinoma. Cell.

[30] Zhu Q, Tao B, Chen H, Shi H, Huang L, Chen J, Hu M, Lo LJ, Peng J (2021). Rcl1 depletion impairs 18S pre-rRNA processing at the A1-site and up-regulates a cohort of ribosome biogenesis genes in zebrafish. Nucleic Acids Res.

[31] Brownstein CA, Smith RS, Rodan LH, Gorman MP, Hojlo MA, Garvey EA, Li J, Cabral K, Bowen JJ, Rao AS (2021). RCL1 copy number variants are associated with a range of neuropsychiatric phenotypes. Mol Psychiatry.

[32] Bernstein KA, Bleichert F, Bean JM, Cross FR, Baserga SJ (2007). Ribosome biogenesis is sensed at the start cell cycle checkpoint. Mol Biol Cell.

[33] Iwanami N, Higuchi T, Sasano Y, Fujiwara T, Hoa VQ, Okada M, Talukder SR, Kunimatsu S, Li J, Saito F (2008). WDR55 is a nucleolar modulator of ribosomal RNA synthesis, cell cycle progression, and teleost organ development. PLoS Genet.

[34] Strezoska Z, Pestov DG, Lau LF (2002). Functional inactivation of the mouse nucleolar protein Bop1 inhibits multiple steps in pre-rRNA processing and blocks cell cycle progression. J Biol Chem.

[35] Pestov DG, Strezoska Z, Lau LF (2001). Evidence of p53-dependent cross-talk between ribosome biogenesis and the cell cycle: effects of nucleolar protein Bop1 on G(1)/S transition. Mol Cell Biol.

[36] Rohrmoser M, Hölzel M, Grimm T, Malamoussi A, Harasim T, Orban M, Pfisterer I, Gruber-Eber A, Kremmer E, Eick D (2007). Interdependence of Pes1, Bop1, and WDR12 controls nucleolar localization and assembly of the PeBoW complex required for maturation of the 60S ribosomal subunit. Mol Cell Biol.

[37] Grimm T, Hölzel M, Rohrmoser M, Harasim T, Malamoussi A, Gruber-Eber A, Kremmer E, Eick D (2006). Dominant-negative Pes1 mutants inhibit ribosomal RNA processing and cell proliferation via incorporation into the PeBoW-complex. Nucleic Acids Res.

[38] Hölzel M, Rohrmoser M, Schlee M, Grimm T, Harasim T, Malamoussi A, Gruber-Eber A, Kremmer E, Hiddemann W, Bornkamm GW (2005). Mammalian WDR12 is a novel member of the Pes1-Bop1 complex and is required for ribosome biogenesis and cell proliferation. J Cell Biol.

[39] Nishimura K, Kumazawa T, Kuroda T, Katagiri N, Tsuchiya M, Goto N, Furumai R, Murayama A, Yanagisawa J, Kimura K (2015). Perturbation of ribosome biogenesis drives cells into senescence through 5S RNP-mediated p53 activation. Cell Rep.

[40] Llovet JM, De Baere T, Kulik L, Haber PK, Greten TF, Meyer T, Lencioni R (2021). Locoregional therapies in the era of molecular and immune treatments for hepatocellular carcinoma. Nat Rev Gastroenterol Hepatol.

[41] Bianco C, Mohr I (2019). Ribosome biogenesis restricts innate immune responses to virus infection and DNA. Elife.

[42] Dersh D, Holly J, Yewdell JW (2021). A few good peptides: MHC class I-based cancer immunosurveillance and immunoevasion. Nat Rev Immunol.

[43] Wei J, Kishton RJ, Angel M, Conn CS, Dalla-Venezia N, Marcel V, Vincent A, Catez F, Ferré S, Ayadi L (2019). Ribosomal proteins regulate MHC Class I peptide generation for immunosurveillance. Mol Cell.

[44] Lu C, Rong D, Zhang B, Zheng W, Wang X, Chen Z, Tang W (2019). Current perspectives on the immunosuppressive tumor microenvironment in hepatocellular carcinoma: challenges and opportunities. Mol Cancer.

[45] Strilic B, Yang L, Albarran-Juarez J, Wachsmuth L, Han K, Muller UC, Pasparakis M, Offermanns S (2016). Tumour-cell-induced endothelial cell necroptosis via death receptor 6 promotes metastasis. Nature.

[46] Jung S, von Thülen T, Yang I, Laukemper V, Rupf B, Janga H, Panagiotidis G-D, Schoen A, Nicolai M, Schulte LN (2020). A ribosomal RNA fragment with 2',3'-cyclic phosphate and GTP-binding activity acts as RIG-I ligand. Nucleic Acids Res.

