# Supplementary figures and images for: Rcl1 suppresses tumor progression of hepatocellular carcinoma: a comprehensive analysis of bioinformatics and in vitro experiments
Source: Cancer Cell Int. 2022 Mar 9;22:114. doi: 10.1186/s12935-022-02533-x (PMC8905783; doi:10.1186/s12935-022-02533-x)

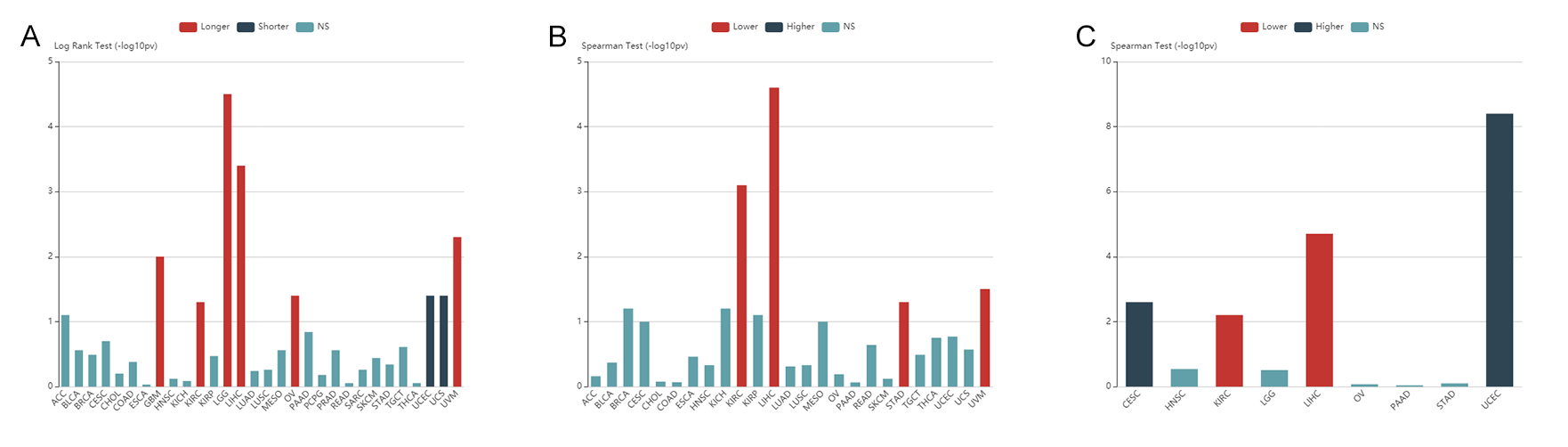

Supplement: Supplementary file 1 — Additional file 1: Fig. S1. The landscape of relationship between Rcl1 mRNA expression and overall survival, tumor stage, histological grade in different types of cancer. (A) Overall survival (B) Tumor stage, (C) Histological grade. Longer (or Shorter): the gene is associated with longer (shorter) survival (Log rank test: p < 0.05); Lower (or Higher): the gene is associated with lower (or higher) stage (Spearman correlation test: p < 0.05); Lower (or Higher): the gene is associated with lower (or higher) stage (Spearman correlation: p < 0.05); NS No significant. [file 12935_2022_2533_MOESM1_ESM.tif]

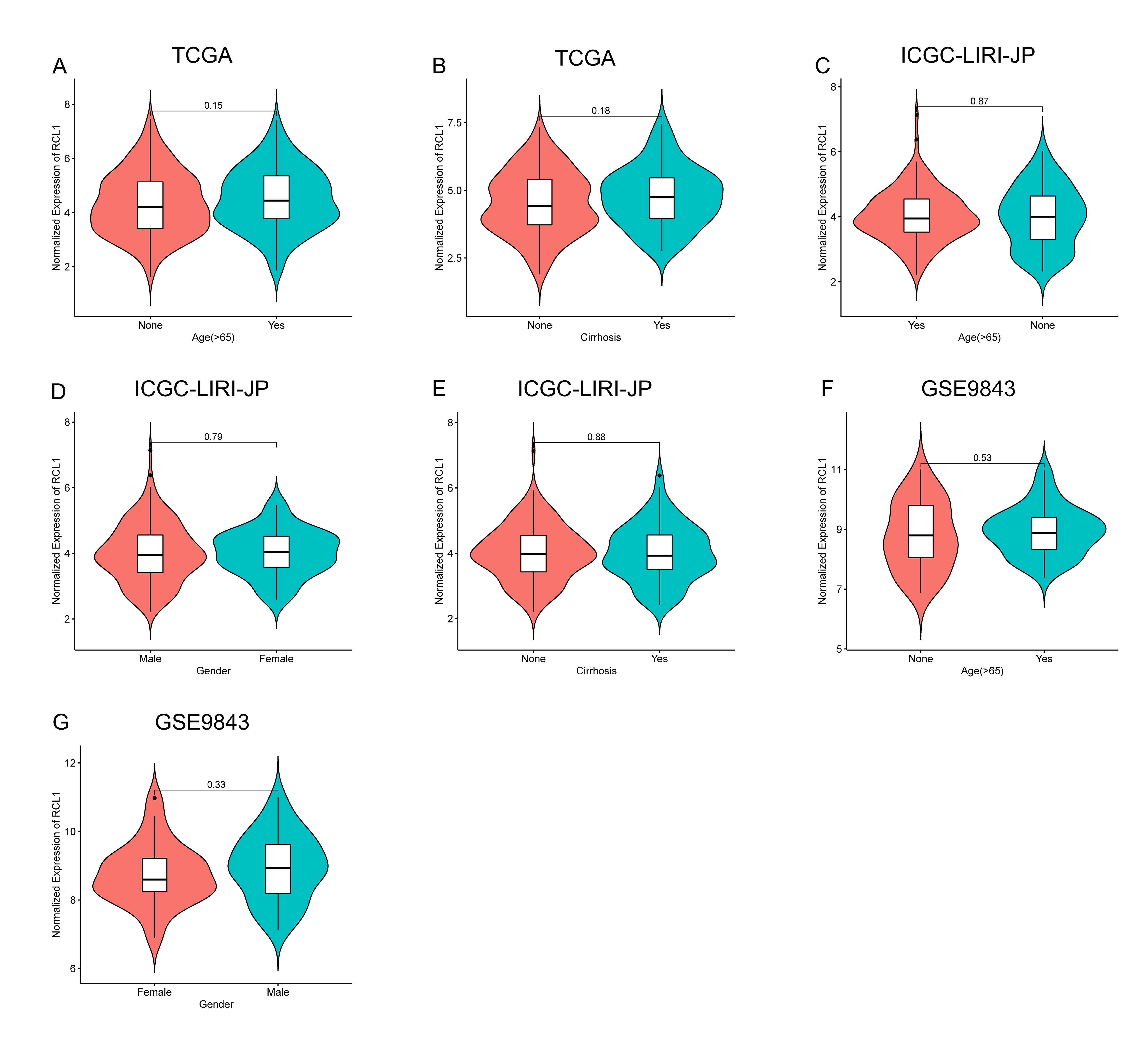

Supplement: Supplementary file 2 — Additional file 1: Fig. S2. Association between RCL1 expression and clinicopathologic characteristics in the HCC cohorts. No significant correlation between RCL1 expression and (A) age (B) cirrhosis in TCGA cohort, (C) age (D) gender (E) cirrhosis in ICGC cohort, (F) age (G) gender in GSE9843 cohort. [file 12935_2022_2533_MOESM2_ESM.tif]
